# Exosomes Derived from Adipose Mesenchymal Stem Cells Carrying miRNA-22-3p Promote Schwann Cells Proliferation and Migration through Downregulation of PTEN

**DOI:** 10.1155/2022/7071877

**Published:** 2022-09-13

**Authors:** Jianqiang Yang, Baoxin Wang, Yating Wang, Chen Feng, Lixiao Chen, Yuying Liu, Xinwei Chen, Pin Dong

**Affiliations:** Department of Otorhinolaryngology and Head and Neck Surgery, Shanghai General Hospital, School of Medicine, Shanghai Jiaotong University, Shanghai, China

## Abstract

Peripheral nerve injury (PNI) is often resulting from trauma, which leads to severe and permanently disability. Schwann cells are critical for facilitating the regeneration process after PNI. Adipose-derived mesenchymal stem cells (ADSCs) exosomes have been used as a novel treatment for peripheral nerve injury. However, the underlying mechanism remains unclear. In this study, we isolated ADSCs and extracted exosomes, which were verified by transmission electron microscopy (TEM), nanoparticle tracking analysis (NTA), and western blot (WB). Cocultured with Dorsal Root Ganglion (DRG) and Schwann cells (SCs) to evaluate the effect of exosomes on the growth of DRG axons by immunofluorescence, and the proliferation and migration of SCs by CCK8 and Transwell assays, respectively. Through exosomal miRNA sequencing and bioinformatic analysis, the related miRNAs and target gene were predicted and identified by dual luciferase assay. Related miRNAs were overexpressed and inhibited, respectively, to clarify their effects; the downstream pathway through the target gene was determined by real-time fluorescence quantitative polymerase chain reaction (RT-qPCR) and WB. Results found that ADSC-exosomes could promote the proliferation and migration of SCs and the growth of DRG axons, respectively. Exosomal miRNA-22-3p from ADSCs directly inhibited the expression of Phosphatase and Tensin Homolog deleted on Chromosome 10 (PTEN), activated phosphorylation of the AKT/mTOR axis, and enhanced SCs proliferation and migration. In conclusion, our findings suggest that ADSC-exosomes could promote SCs function through exosomal miRNA-22-3p, which could be used as a therapeutic target for peripheral nerve injury.

## 1. Introduction

The peripheral nerve connects the central nerve to the muscles, bones, and visceral organs. Peripheral nerve injuries (PNIs) often result in persistent dysfunction of innervated tissues. PNIs occur secondary to trauma, tumors or inflammation, with an annual incidence of approximately 13–23 per 100,000 individuals [1]. Trauma is the primary cause, and approximately 3% of trauma events are accompanied by PNIs [2]. Because of inflammation and scar formation, recovery from PNIs is often limited by the type and severity of injury. Currently, autologous nerve transplantation is the standard treatment for complete amputation, a type of severe injury. However, this method requires sacrificing the normal donor nerves, and simple end-to-end nerve suturing may result in the wrong direction of sensory and motor nerve regeneration [3]. This results in an unsatisfactory outcome; therefore, new treatments for PNIs are needed.

With the development of regenerative medicine, mesenchymal stem cells (MSCs) have increasingly been used to treat various diseases and injuries. MSCs can promote the repair of nerve injuries [4], which may occur by two mechanisms. First, MSCs can differentiate into Schwann-like cells or promote the massive proliferation of Schwann cells (SCs), thus providing physical support for the regeneration and extension of nerve axons [5]. In addition, MSCs can secrete neurotrophic factors such as BDNF, GDNF, and NGF, which promote nerve repair [6]. Nevertheless, there are still some limitations to the direct use of MSCs to treat PNIs, such as unavoidable immunogenicity and ethical issues that limit their clinical application.

Exosomes are a class of membranous vesicles that are approximately 30–150 nm in size. They are produced by most cells, including MSCs, tumor cells, immune cells, and nerve cells [7–9]. Exosomes play an important role in intercellular communication. They can transport a variety of bioactive substances, including lipids, proteins, and nucleic acids, to recipient cells for transmission of biological information. Studies have shown that miRNAs in exosomes can bind to target genes associated with various cellular pathways, including angiogenesis, cell transport, apoptosis, and protein cleavage [10, 11]. In the nervous system, MSC-derived exosomes also play an important role in nerve regeneration [12]. A study showed that adipose-derived MSCs (ADSCs) exosomes can promote the proliferation and migration of Schwann cells [13]; however the mechanism is not clear. Moreover, the role of miRNAs in ADSC-exosomes (ADSC-Exos) remains unknown.

In the present study, exosomes were extracted from ADSCs and cocultured with SCs for exosomal miRNA sequencing to identify relevant miRNAs, as well as the downstream signaling pathways and mechanisms associated with their effects.

## 2. Materials and Methods

### 2.1. Isolation of ADSCs

Sprague-Dawley rats were purchased from Shanghai SLAC Animal Company. All animal experiments were approved by the Ethics Committee of Shanghai General Hospital and conducted strictly in accordance with the institutional guidelines for the care and use of laboratory animals. Four-week-old SD rats were anesthetized, and adipose biopsies were obtained from inguinal subcutaneous tissue and cut into small pieces using microscissors. Added 0.1% collagenase I (SCR103; Sigma-Aldrich) to digest at a shaker (37°C for 1 hour). Digested adipose tissue was centrifuged at 2000 × rpm for 10 min. After filtering through a 70 *μ*m mesh filter, cells were cultured at 37°C in 5% CO_2_ humidified conditions in DMEM medium (11965092; Gibco) with 10% fetal bovine serum (FBS) (10082147; Gibco), and 1% penicillin/streptomycin (15070063; Gibco) [14]. The medium was replaced every two days and passaged when 90% fusion was reached.

### 2.2. Multipotent Differentiation of ADSCs

When the ADSCs reached 70% confluence, the medium was replaced with osteogenic induction medium supplemented with L-glutamine, dexamethasone, ascorbic acid, and *β*-glycerophosphate. The medium was changed every 3 days according to the growth conditions. After 4 weeks, the cells were stained with Alizarin Red S stain [15]. When ADSCs reached 100% confluence, the medium was replaced with adipogenic induction culture medium consisting of DMEM with insulin, dexamethasone, indomethacin, and 3-isobutyl-1-methylxanthine. The medium was replaced every three days for 14 days. Oil Red O staining was performed to detect intracellular lipid vacuoles characteristic of adipocytes [16].

### 2.3. Flow Cytometric Analysis

Cells were incubated with a specific monoclonal antibody conjugated with phycoerythrin (PE) in 200 *μ*l PBS for 30 min in the dark at 4°C and then analyzed by flow cytometry. Antibodies against PE anti-CD11b/c (201807; BioLegend), PE anti-CD29 (102207; BioLegend), PE anti-CD45 (202207; BioLegend), and PE anti-CD90 (202523; BioLegend) were used. PE anti-mouse IgG1 (4066607; BioLegend) was used as the control.

### 2.4. Extraction of Exosomes and miRNA

ADSCs were cultured in DMEM containing 10% exosomes-free FBS (180625; SBI) for 48 h. The medium was then collected and centrifuged at 3000 × *g* for 10 min at 4°C. The supernatant was collected, transferred to a new tube, and placed on ice. Exosomes and miRNAs were extracted using an exoRNeasy Midi Kit (77144; QIAGEN), according to the manufacturer's protocol.

### 2.5. Identification of Exosomes

The morphology of exosomes was examined by transmission electron microscopy (TEM). Exosomes were fixed in 2% paraformaldehyde (PFA)-cacodylate buffer and loaded onto copper grids covered with formvar for 30 min. Grids were washed and contrasted in 4% uranyl acetate for 5 min before being examined by TEM (FEI TECNAI G2 spititi FEI, 120 KV). The particle size of the exosomes was determined using nanoparticle tracking analysis (NTA) with Zeta View PMX 110 (Particle Metrix, Meerbusch) and the corresponding software Zeta View 8.04.02. The exosomal characteristic markers rabbit pAb CD9 (A1703; ABclonal), rabbit mAb ALIX (ab275377; Abcam), and mouse mAb GAPDH (ab8245; Abcam) were analyzed by western blotting [17].

### 2.6. Internalization Assays of Exosomes

ADSC-Exos were labeled with PKH67 (MINI67-1KT; Sigma-Aldrich) according to the manufacturer's instructions. Schwann cells were seeded in confocal dishes and coculture with PKH67 labeled ADSC-Exos. After 24 h, Schwann cells were fixed with 4% formaldehyde and stained with Actin-Tracker Red-594 (C2205S; Beyotime) and DAPI (C1005; Beyotime). Finally, cells were visualized using a laser scanning confocal microscope.

### 2.7. Isolation and Culture of DRG

Three-week-old SD rats were sacrificed by decapitation with guillotine after anesthesia. The body trunk of the rat was isolated between the forelimb and femur, and the bilateral lumbar DRGs were collected in a 35-mm culture dish with 2 ml of iced Leibovitz's L-15 medium (11415064; Gibco) [18]. Added 2 ml of prewarmed digestive system (DNase 0.1 mg/ml; Trypsin 0.4 mg/ml; Collagenase I 1 mg/ml) and digested the DRGs for 40 minutes in an incubator (37°C, 5%CO_2_). After digestion was stopped, the cell suspension was filtered through a 100 *μ*m membrane filter and plated onto a poly D-lysine-coated confocal dish with DMEM complete medium. Approximately 1 d later, the Neurobasal complete medium with the addition of 10 *μ*m cytarabine (Ara-C) was replaced. After 3 days, ADSC-Exos were added to the DRGs. The control group contained no exosomes.

### 2.8. Immunofluorescence Staining

The cells were then fixed with 4% paraformaldehyde for 30 min. After permeability and closure, the samples were incubated with primary antibodies (1 : 300, Cell Signaling Technology) overnight at 4°C and then treated with the corresponding fluorescence labeled secondary antibodies (1 : 300; Invitrogen). The results were observed using a laser scanning confocal microscope.

### 2.9. Cell Counting Kit-8 (CCK-8) Assays

Cell proliferation was detected using a Cell Counting Kit-8 kit (HY-K0301; MCE). After coculture with ADSC-Exos for 48 h, cells were seeded in 96-well plates and incubated for 24-72 h after treatment. Each well received a 10 *μ*l CCK-8 reagent and was cultured in a cell incubator for 1 h. Optical density (OD) at 450 nm was measured using a microplate reader.

### 2.10. Transwell Assays

Migration was evaluated using a 24-well Transwell system (3422; Corning). The cells were then paved in the apical chamber in serum-free medium. The basolateral chamber was filled with a medium supplemented with 10% exosomes-free FBS as a chemical attractant. After 48 h of incubation, the membrane was stained with 0.1% crystal violet. The cells were observed under an inverted microscope.

### 2.11. miRNA Sequencing of Exosomes and Bioinformatic Analysis

Total RNA from ADSC-Exos and SC-Exos was isolated for miRNA analysis using the mirVana™ miRNA Isolation Kit (AM1560; Ambion) according to the manufacturer's protocol. Reverse transcription, library construction, miRNA sequencing, and analysis were conducted by OBIO Biotech Company. Criteria applied to select differentially expressed miRNAs were *p* < 0.05 and fold-change ≥2 or ≤0.5. The potential binding sites of the miRNAs were predicted using the online software TargetScan (http://www.Targetscan.org/vert_72/).

### 2.12. Dual-Luciferase Reporter Assays

The 3′-UTR of the PTEN fragment containing wild-type or mutant binding sites for miR-22-3p was cloned into the pmirGlO luciferase reporter vector (Asia Vector Biotech) to generate the wild-type or mutant plasmids, respectively. miRNA-NC or miR-22-3p was transfected with luciferase reporter plasmids into Schwann cells. After 72 h, luciferase activity was evaluated using a dual-luciferase assay kit (E1910; Promega). All primers used are presented in Supplementary S1.

### 2.13. Quantitative Real-Time Polymerase Chain Reaction

Total RNA was isolated using the miRNeasy Mini Kit (217004, QIAGEN), according to the manufacturer's protocol. The HyperScript^®^ RT Supermix Reagent Kit (R202, NovaBio) and HyperScript™ miRNA 1st Strand cDNA synthesis kit (R601, NovaBio) were used to reverse transcribe mRNA and miRNA into cDNA. Real-time qPCR was performed on the StepOnePlus™ platform (Applied Biosystems) using a SYBR^®^ qPCR Mix kit (Q204, NovaBio). Primers and reaction conditions are provided in Supplementary S2. The relative expression levels of the target genes were calculated using the 2 −  ^ΔΔCt^ method and normalized to GAPDH or U6.

### 2.14. Western Blot

RIPA lysis buffer (89900, Thermo Fisher) was used to lyse the samples, which were then centrifuged at 12000 g for 20 min at 4°C. The protein concentration in the supernatant was calculated using a BCA protein assay kit (P0012; Beyotime). The samples were loaded onto an SDS-PAGE gel, electrophoresed, and transferred to the PVDF membrane. The membranes were incubated with primary antibodies (1 : 1000; CST and ABclonal) overnight at 4°C and with the corresponding secondary antibodies (1 : 20000; Proteintech) at room temperature for 1 h. Immunoblots were observed with an enhanced chemiluminescence reagent kit (P2300; NCM).

### 2.15. Statistical Analysis

All data are expressed as the mean ± SD. Comparisons between two groups were evaluated using an unpaired Student's *t*-test. One-way ANOVA with a Bonferroni posthoc test was used for groups ≥3. Statistical significance was set at *p* < 0.05. Statistical analysis was performed using the GraphPad Prism 8.0.2 software.

## 3. Results

### 3.1. Identification of ADSCs

**Figure 1:**
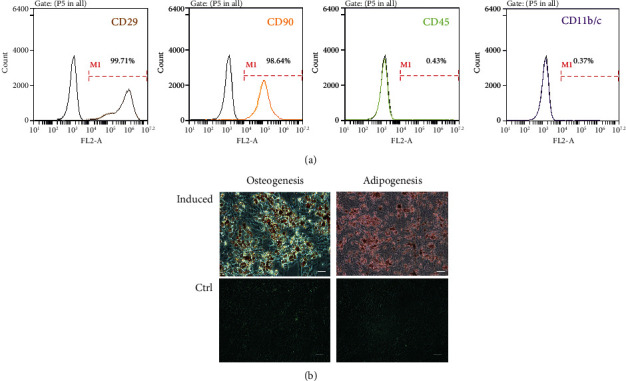
Characterization of ADSCs. (a) Flow cytometric analysis of surface markers of ADSCs. ADSCs were positive for MSC markers including CD29 (99.71%) and CD90 (98.64%) and were negative for CD45 (0.43%) and CD11b/c (0.37%). (b) Representative images of adipogenic differentiation and osteogenic differentiation. The visual field was filled with red lipid droplets stained with Oil Red O solution in images of adipogenic differentiation. Calcium nodules with burrs were formed in images of osteogenic differentiation, which were stained red by Alizarin Red S solution (scale bar: 100 *μ*m). CTRL, Control group; osteogenesis, osteogenic induction group; adipogenesis, adipogenic induction group; ADSCs, Adipose-derived mesenchymal stem cells.

ADSCs were isolated from SD rats and passaged to the 3rd generation. Flow cytometry analysis revealed that the ADSCs were positive for MSC markers, including CD29 (99.71%) and CD90 (98.64%), and negative for CD45 (0.43%) and CD11b/c (0.37%) (Figure 1(a)). To verify the multipotent differentiation ability, we conducted adipogenic and osteogenic experiments, and the results indicated that they exhibited significant differentiation potential (Figure 1(b)).

### 3.2. Characterization and Internalization of ADSC-Exos

**Figure 2:**
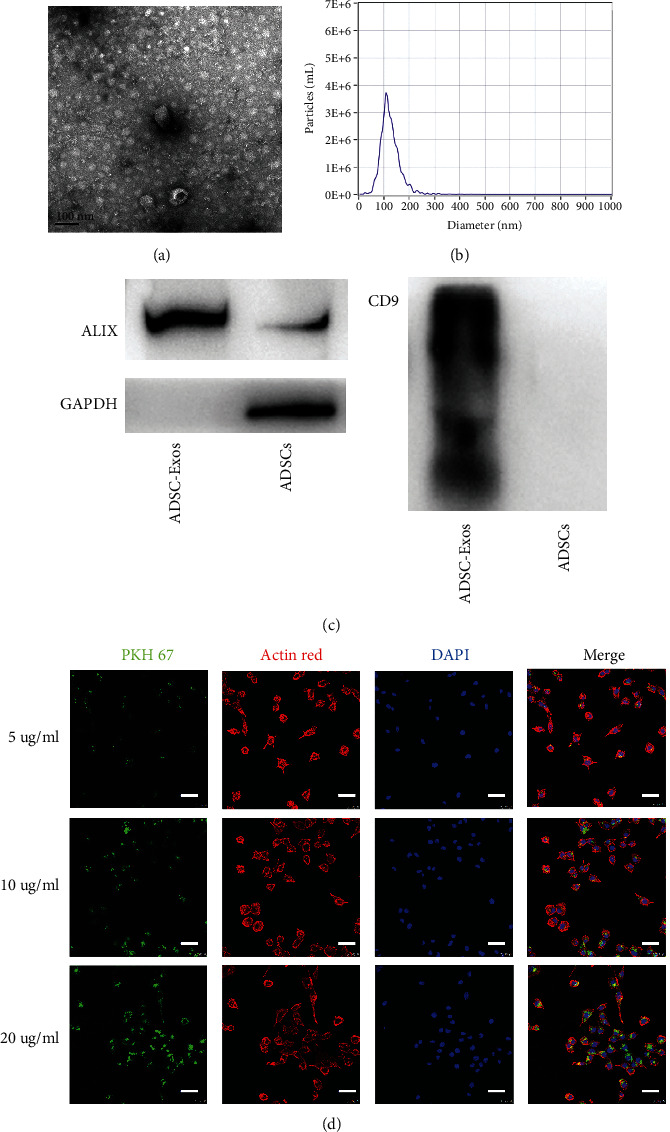
Characterization of exosomes from ADSCs. (a) Representative ultrastructure of ADSC-Exos by TEM. (b) The size distribution profile of ADSC-Exos by NTA. (c) Detection of exosomal markers (ALIX and CD9) and GAPDH by western blot.(d) The internalization of ADSC-Exos by SCs. Confocal images showing the SCs incubated with 5, 10, and 20 *μ*g/ml PKH67-labeled ADSC-Exos for 24 hour (scale bar : 100 *μ*m). TEM, Transmission Electron Microscope; NTA, Nanoparticle Tracking Analysis; DAPI, 4′-6-diamidino-2-phenylindole; PKH67, Green Fluorescent Cell Linker; Actin Red, phalloidin-Alexa Fluor 594; ADSCs, Adipose-derived mesenchymal stem cells; ADSC-Exos, the exosomes extracted from Adipose-derived mesenchymal stem cells.

Next, we characterized ADSC-Exos by isolating exosomes from the conditioned medium of the ADSCs. First, exosomes were resuspended in PBS and characterized using transmission electron microscopy (TEM). ADSC-Exos exhibited the characteristic structure of exosomes (Figure 2(a)). Second, we used NTA to confirm the size and concentration of released exosomes. The mean particle diameter was 110.2 nm, with a concentration of 1.5 × 10^10^ particles/ml (Figure 2(b)). Finally, western blot analysis of the purified exosomes revealed the expression of exosomal markers CD9 and ALIX, as well as the negative marker GAPDH (Figure 2(c)). Exosomes were labeled with PKH67 to determine whether ADSC-Exos were taken up by Schwann cells. We cocultured different concentrations (5, 10, and 20 *μ*g/ml) of ADSC-Exos with Schwann cells. After 24 h, confocal imaging revealed the presence of PKH67 spots in the recipient cells, indicating that the labeled exosomes released by ADSCs could be delivered to Schwann cells, and the uptake efficiency was concentration-dependent (Figure 2(d)).

### 3.3. Effects of ADSC-Exos on DRG Neurite Growth and SCs Proliferation and Migration

**Figure 3:**
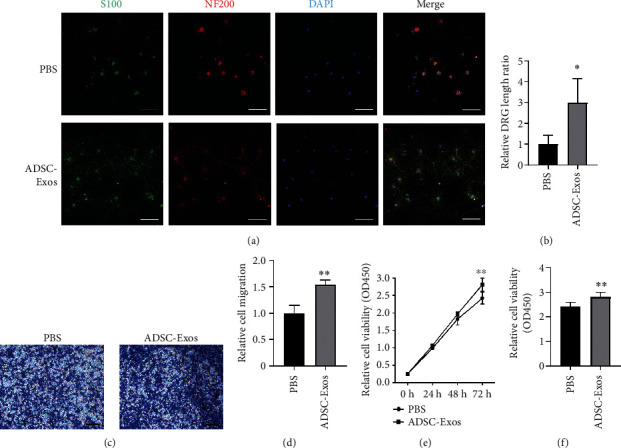
ADSC-Exos can enhance DRG neurite outgrowth and proliferation and migration of SCs. (a) DRG were stained with S100 antibody (green) and NF200 antibody (red). (b) Statistical analysis of length of DRG neurite. (c–d) SCs migration after cocultured with ADSC-Exos were measured by the Transwell assays. (e–f) SCs proliferation after cocultured with ADSC-Exos were measured by the CCK-8 assays. Scale bar : 100 *μ*m. Data are expressed as means ± SD. Statistical significance was obtained with Student's *t*-test, ^∗^ means *p* < 0.05; ^∗∗^ means *p* < 0.01. Control group added PBS; ADSC-Exos, the exosomes extracted from Adipose-derived mesenchymal stem cells.

We isolated rat primary DRGs to determine the effects of ADSC-Exos on neurite growth. After coculturing with ADSC-Exos for 3 days, immunofluorescence staining was performed to evaluate the effect of ADSC-Exos on DRG neurite growth compared with the PBS-treated control group (Figure 3(a)). The DRGs clearly developed neurites in the ADSC-Exos group, and the length of the longest neurite was significantly increased compared with that of the control group (Figure 3(b)). Next, we determined whether ADSC-Exos affected the migration and proliferation of Schwann cells. The migration assay revealed that ADSC-Exos markedly enhanced cells migration at 48 h (Figures 3(c) and 3(d)). In addition, the CCK-8 assay indicated that ADSC-Exos promoted the proliferation of Schwann cells, and there was a statistical difference between the two groups at 72 h (Figure 3(e) and 3(f)).

### 3.4. Exosomal miRNA Sequencing

**Figure 4:**
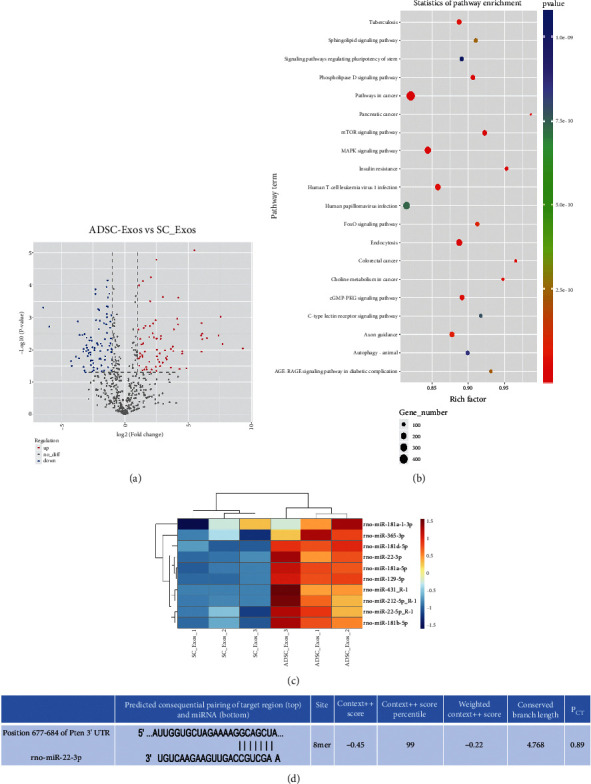
The miRNA expression profile between ADSC-Exos and SC-Exos. (a) The distribution of the differentially expressed miRNAs of ADSC-Exos and SC-Exos in a volcano plot. (b) The top 20 significant KEGG pathway terms sorted by rich factor. (c) Heatmap of 10 miRNAs with high expression in mTOR signaling pathway predicted by sequencing and TargetScan sofeware. (d) Binding sites of miR-22-3p and Pten predicted by TargetScan software. ADSC-Exos, the exosomes extracted from Adipose-derived mesenchymal stem cells; SC-Exos, the exosomes extracted from Schwann cells; miR, miRNA.

miRNA sequencing results revealed that 124 miRNAs were upregulated in ADSC-Exos compared to SC-Exos (*p* < 0.05) (Supplementary S3). The distribution of the differentially expressed miRNAs of ADSC-Exos compared to SC-Exos is shown in a volcano plot, as shown in Figure 4(a). Using the KEGG database, the target genes were found to be enriched in several signaling pathways, in particular, 143 miRNAs were enriched in the mTOR signaling pathway (Figure 4(b)). We selected ten miRNAs with high different expression (*p* < 0.05) in sequencing and high score in the TargetScan software as candidates (Figure 4(c)). The results indicated that miR-22-3p has significant potential, and Pten (tensin homolog deleted on chromosome 10) was predicted to be its target gene (Figure 4(d)).

### 3.5. miR-22-3p Directly Targets Pten in SCs

**Figure 5:**
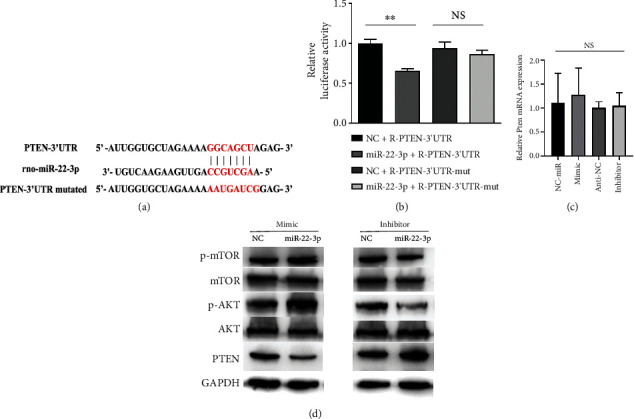
miR-22-3p targets PTEN and activates the AKT/mTOR signaling pathway. (a) The predicted binding sites of miR-22-3p within the 3′-UTR of Pten. (b) Dual-luciferase reporter assays showed miRNA-22-3p cotransfected with wild-type or mutation Pten 3′-UTR in SCs. (c) Pten mRNA expression in SCs after the treatment with miR-22-3p mimic, miR-22-3p inhibitor, and NC. (d) Effects of miR-22-3p mimic and miR-22-3p inhibitor on the protein expressions of AKT/mTOR pathway compare with control group. Data are expressed as means ± SD. Statistical significance was obtained with unpaired Student's *t* test or one way ANOVA. ^∗∗^ means *p* < 0.01; NS means no significant. ADSC-Exos, the exosomes extracted from Adipose-derived mesenchymal stem cells; SCs, Schwann cells; miR, miRNA.

To explore the mechanism of miR-22-3p regulation, a bioinformatic analysis was performed, which revealed that the target site was located at 674-681 bp in the 3′UTR of Pten. Therefore, we designed a luciferase vector containing a wild-type or mutant Pten 3′-UTR (Figure 5(a)). To verify whether miR-22-3p directly targets the 3′ UTR of Pten directly, a dual luciferase reporter assay was conducted. The results showed that the luciferase activity of the Pten WT 3′UTR was significantly reduced after cotransfection with miR-22-3p compared to that in the NC group. In contrast, no significant direct interaction was observed between miR-22-3p and the vector containing the Pten MUT 3′UTR (Figure 5(b)).

### 3.6. ADSC-Exos Promote SC Proliferation and Migration via miRNA-22-3p

**Figure 6:**
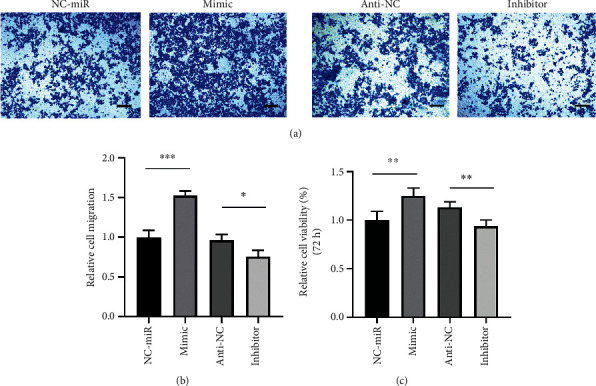
miR-22-3p promotes Schwann cells migration and proliferation in vitro. (a–b) The effect of miR-22-3p on the migration of Schwann cells was assessed by a Transwell assays, scale bars : 100 um. (c) Statistical analysis of CCK-8 assays at 72 h. Results from 3 independent experiments with duplicates. Data are expressed as means ± SD. Statistical significance was obtained with unpaired Student's *t* test. ^∗^ means *p* < 0.05; ^∗∗^ means *p* < 0.01; ^∗∗∗^ means *p* < 0.001. ADSC-Exos, the exosomes extracted from Adipose-derived mesenchymal stem cells; SCs, Schwann cells; miR, miRNA.

To reveal the downstream regulatory effects of miR-22-3p, we synthesized a mimic and an inhibitor of miR-22-3p, as well as a negative control, which were transfected into Schwann cells to determine the relationship between miR-22-3p and Pten whether it regulates Schwann cells proliferation and migration. The results demonstrated that the Pten mRNA levels were unchanged in each group (Figure 5(c)); however, the expression of PTEN protein was downregulated by the miR-22-3p mimic but upregulated by the inhibitor compared to the NC group. The activity of the AKT/mTOR pathway was evaluated based on exosomal miRNA sequencing results. The miR-22-3p mimic reduced the expression of PTEN and positively regulated the AKT/mTOR pathway, where the miR-22-3p inhibitor had a negative effect on this pathway (Figure 5(d)). Accordingly, we performed transwell and CCK-8 assays. Upregulation of miR-22-3p significantly increased cell migration, whereas the miR-22-3p inhibitor decreased cell motility in Schwann cells (Figure 6(a) and 6(b)). The CCK-8 assay revealed that the mimic also promoted the proliferation of Schwann cells, whereas the inhibitor had a negative effect (Figure 6(c)). Overall, these results indicate that exosomal miR-22-3p promotes proliferation and migration of Schwann cells by activating the AKT/mTOR signaling pathway.

## 4. Discussion

Although the vast majority of PNIs do not threaten the lives of patients, persistent damage brings not only long-term physical and mental harm to patients but also a heavy burden on families and society [19]. Following PNI, massive proliferation of SCs is triggered to form Bungner bands, which provide a large number of nutritional substrates for axon growth, including a considerable amount of extracellular matrix proteins and a variety of neurotrophic factors [20]. Additionally, SCs form a “myelin scaffold” to support axons extending distally at an average speed of 1 mm/day [21, 22]. These findings demonstrate that changes in the proliferative and migratory abilities of SCs during the repair of PNIs can influence the regeneration of nerve axons.

In the present study, we selected ADSCs as subjects because they have the advantage of minimal tissue damage after sampling, mass availability, repeatability, and high clinical value [23]. We successfully isolated primary ADSCs, identified the surface antigens using flow cytometry, and confirmed their adipogenic and osteogenic abilities through direction-induced differentiation. ADSCs promote the repair of PNIs; however, it has been reported that a paracrine mechanism is involved in MSC-mediated tissue repair, whereas direct differentiation of stem cells is weak [24, 25]. Considering the limitations of stem cell applications and the fact that the paracrine function of stem cells can be mediated by exosomes, the use of exosomes to replace stem cells for direct treatment has become a feasible alternative method.

By coculturing rat ADSC-Exos with SCs, we found that ADSC-Exos not only promoted SC proliferation but also enhanced their migratory ability, which was demonstrated for the first time. As mentioned above, SC proliferation results in the formation of new myelin channels that promote axonal regeneration. The results of our DRGs' experiment confirmed this hypothesis. Some researchers cocultured conditioned medium from mouse ADSCs with SCs and DRGs and found that the proliferation of SCs were enhanced as well as the growth of DRG axons [26]; the underlying reason may be that exosomes in the conditioned medium are responsible for the above effects. Another study reported that human ADSC-Exos significantly promoted SC proliferation, migration, and myelination [13], which are consistent with the results of this study.

However, the mechanism by which ADSC-Exos promote SC proliferation and migration remains unclear. Exosomes can carry a variety of bioactive substances and mediate intercellular signal transduction in MSC-derived exosomes mediate intercellular signal transduction [12, 27]. In the peripheral nervous system, exosomes can transport miRNAs to axons [28]. Therefore, exosomal miRNAs may be key elements in the effects of exosomes, and identifying the relevant miRNAs in exosomes is a prerequisite to clarify the role of exosomes in promoting peripheral nerve regeneration and postinjury repair. We speculated that miRNAs in rat ADSC-Exos may play an important role in promoting the proliferation and migration of SCs. To further explore the role of miRNAs, we performed exosomal miRNA sequencing and subsequent bioinformatic analysis.

Sequencing results revealed that 124 miRNAs were highly expressed in ADSCs. Based on KEGG pathway analysis, 143 miRNAs were enriched in the mTOR pathway. To identify the miRNAs associated with SC proliferation and migration, miR-181a-3p, miR-365-3p, miR-22-3p, miR-22-5p, miR-181a-5p, miR-181b-5p, miR-129-5p, miR-431, miR-212-5p, and miR-365-3p were selected for further bioinformatic analysis. TargetScan database search revealed that miR-22-3p is very likely to bind directly to phosphatase and tensin homolog deleted on chromosome 10 (PTEN). In addition, overexpression of miR-22-3p in HK-2 cells can directly inhibit PTEN [29]. Consequently, we focused on miR-22-3p and examined the target gene, Pten, of miR-22-3p in the Akt/mTOR pathway. PTEN is an inhibitor of the Akt/mTOR signaling pathway. Following PTEN downregulation, Akt is activated and phosphorylated to promote the activation of downstream mTOR. As an important signal transduction pathway, the PI3K/Akt/mTOR pathway plays an important biological role in cell growth, survival, proliferation, apoptosis, angiogenesis, and autophagy [30]. Conditional knockout of PTEN gene can promote regeneration of corticospinal tract (CST) axons and recovery of motor function in mice [31]. Another study also showed that PTEN knockout promotes CST regeneration, and the mechanism may involve increased mTOR activity, thus enhancing the regeneration ability of cortical neurons [32]. This indicates that repair of the central nervous system can be promoted by enhancing Akt/mTOR signaling activity. In our previous study, we found that negative regulation of PTEN activates the PI3K/Akt signaling pathway and promotes repair of the recurrent laryngeal nerve [33]. Moreover, the results of this study further confirmed that miR-22-3p directly binds to PTEN and promotes the phosphorylation of the downstream Akt/mTOR axis by inhibiting PTEN expression, thus promoting the proliferation and migration of SCs. Regarding future clinical translation for the treatment of PNIs, ADSCs may be extracted from the adipose tissue of patients and exosomes overexpressing miR-22-3p may be obtained after rapid amplification. Alternatively, exosomes may be obtained by overexpression of miR-22-3p and transplanted into the site of PNIs combined with nerve conduits.

## 5. Conclusion

In this study, we confirmed that ADSC-Exos promoted the proliferation and migration of SCs and the growth of DRG axons. Through exosomal sequencing and bioinformatic analysis, miR-22-3p was identified and its role in activating the Akt/mTOR axis by inhibiting PTEN was confirmed. These results provide a new strategy for the treatment of PNIs with stem cell-derived exosomes as a novel treatment method for PNIs.

## Data Availability

All data used to support the findings of this study are available from the corresponding author upon request.

## Supplementary Materials

Supplementary Materials Supplementary S1 Primers used in Dual-Luciferase Reporter Assays. Supplementary S2: Reaction conditions and primers used in reverse transcription and RT-qPCR. Supplementary S3: The miRNA different expression between ADSC-Exos and SC-Exos, heat map showing the dominant cluster pattern of miRNAs under p <0.05. Red signals and blue signals represent upregulated and downregulated expression, respectively.

## References

[1] Sabatier M. J., English A. W. (2015). Pathways mediating activity-induced enhancement of recovery from peripheral nerve. *Exercise and Sport Sciences Reviews*.

[2] Modrak M., Talukder M. A. H., Gurgenashvili K., Noble M., Elfar J. C. (2020). Peripheral nerve injury and myelination: potential therapeutic strategies. *Journal of Neuroscience Research*.

[3] Taylor C. A., Braza D., Rice J. B., Dillingham T. (2008). The incidence of peripheral nerve injury in extremity trauma. *American Journal of Physical Medicine & Rehabilitation*.

[4] Jiang L., Zheng Y., Chen O., Chu T., Ding J., Yu Q. (2016). Nerve defect repair by differentiated adipose-derived stem cells and chondroitinase ABC-treated acellular nerves. *The International Journal of Neuroscience*.

[5] Reid A. J., Sun M., Wiberg M., Downes S., Terenghi G., Kingham P. J. (2011). Nerve repair with adipose-derived stem cells protects dorsal root ganglia neurons from apoptosis. *Neuroscience*.

[6] Fu X. M., Wang Y., Fu W. L. (2019). The combination of adipose-derived Schwann-like cells and acellular nerve allografts promotes sciatic nerve regeneration and repair through the JAK2/STAT3 signaling pathway in rats. *Neuroscience*.

[7] Zheng L., Cui H. F. (2010). Use of chitosan conduit combined with bone marrow mesenchymal stem cells for promoting peripheral nerve regeneration. *Journal of Materials Science. Materials in Medicine*.

[8] Hill A. F. (2019). Extracellular vesicles and neurodegenerative diseases. *The Journal of Neuroscience*.

[9] Hong P., Yang H., Wu Y., Li K., Tang Z. (2019). The functions and clinical application potential of exosomes derived from adipose mesenchymal stem cells: a comprehensive review. *Stem Cell Research & Therapy*.

[10] Zhang B., Shen L., Shi H. (2016). Exosomes from human umbilical cord mesenchymal stem cells: identification, purification, and biological characteristics. *Stem Cells International*.

[11] Eirin A., Ferguson C. M., Zhu X. Y. (2020). Extracellular vesicles released by adipose tissue-derived mesenchymal stromal/stem cells from obese pigs fail to repair the injured kidney. *Stem Cell Research*.

[12] Zhang B., Wu X., Zhang X. (2015). Human umbilical cord mesenchymal stem cell exosomes enhance angiogenesis through the Wnt4/*β*-catenin pathway. *Stem Cells Translational Medicine*.

[13] Chen J., Ren S., Duscher D. (2019). Exosomes from human adipose-derived stem cells promote sciatic nerve regeneration via optimizing Schwann cell function. *Journal of Cellular Physiology*.

[14] González-Cubero E., González-Fernández M. L., Gutiérrez-Velasco L., Navarro‐Ramírez E., Villar‐Suárez V. (2021). Isolation and characterization of exosomes from adipose tissue-derived. *Journal of Anatomy*.

[15] You W. L., Xu Z. L. (2021). Curculigoside promotes osteogenic differentiation of ADSCs to prevent. *Journal of Orthopaedic Surgery and Research*.

[16] Priyadarshini P., Samuel S., Kurkalli B. G. (2021). In vitro comparison of adipogenic differentiation in human adipose-derived stem. *Indian Journal of Plastic Surgery*.

[17] Wu X., Showiheen S. A. A., Sun A. R. (2019). Exosomes extraction and identification. *Methods in Molecular Biology*.

[18] Shekarabi M., Robinson J. A., Burdo T. H. (2021). Isolation and culture of dorsal root ganglia (DRG) from rodents. *Methods in Molecular Biology*.

[19] Rosén B., Vikström P., Turner S. (2015). Enhanced early sensory outcome after nerve repair as a result of immediate post-operative re-learning: a randomized controlled trial. *Journal of Hand Surgery*.

[20] Jessen K. R., Mirsky R., Lloyd A. C. (2015). Schwann cells: development and role in nerve repair. *Cold Spring Harbor Perspectives in Biology*.

[21] García-Medrano B., Mesuro Domínguez N., Simón Pérez C. (2017). Reparacion de lesiones en nervios mediante el implante de protesis obtenidas de segmentos acelulares de nervio isogenico. *Journal of Orthopaedics Surgery and Traumatology*.

[22] Sand J. P., Park A. M., Bhatt N. (2016). Comparison of conventional, revascularized, and bioengineered methods of recurrent laryngeal nerve reconstruction. *JAMA Otolaryngology–Head & Neck Surgery*.

[23] An Y., Lin S., Tan X. (2021). Exosomes from adipose-derived stem cells and application to skin wound healing. *Cell Proliferation*.

[24] Razavi S., Jahromi M., Vatankhah E., Seyedebrahimi R. (2021). Differential effects of rat ADSCs encapsulation in fibrin matrix and combination delivery of BDNF and gold nanoparticles on peripheral nerve regeneration. *BMC Neuroscience*.

[25] Nakajima T., Tada K., Nakada M., Matsuta M., Tsuchiya H. (2021). Facilitatory effects of artificial nerve filled with adipose-derived stem cell sheets on peripheral nerve regeneration: an experimental study. *Journal of Orthopaedic Science*.

[26] Sowa Y., Imura T., Numajiri T., Nishino K., Fushiki S. (2012). Adipose-derived stem cells produce factors enhancing peripheral nerve regeneration: influence of age and anatomic site of origin. *Stem Cells and Development*.

[27] Zhou S., Ding F., Gu X. (2016). Non-coding RNAs as emerging regulators of neural injury responses and regeneration. *Neuroscience Bulletin*.

[28] Hofer H. R., Tuan R. S. (2016). Secreted trophic factors of mesenchymal stem cells support neurovascular and musculoskeletal therapies. *Stem Cell Research & Therapy*.

[29] Wang X., Wang Y., Kong M., Yang J. (2020). MiR-22-3p suppresses sepsis-induced acute kidney injury by targeting PTEN. *Bioscience Reports*.

[30] Yang H. L., Tsai Y. C., Korivi M., Chang C. T., Hseu Y. C. (2017). Lucidone promotes the cutaneous wound healing process *via* activation of the PI_3_K/AKT, Wnt/ *β*-catenin and NF-*κ*B signaling pathways. *Biochim Biophys Acta Mol Cell Res*.

[31] Danilov C. A., Steward O. (2015). Conditional genetic deletion of PTEN after a spinal cord injury enhances regenerative growth of CST axons and motor function recovery in mice. *Experimental Neurology*.

[32] Liu K., Lu Y., Lee J. K. (2010). PTEN deletion enhances the regenerative ability of adult corticospinal neurons. *Nature Neuroscience*.

[33] Xie J., Jin B., Li D. W. (2015). Effect of laminin-binding BDNF on induction of recurrent laryngeal nerve regeneration by miR-222 activation of mTOR signal pathway. *American Journal of Translational Research*.

