# Supplementary material for: Exosomes Derived from Adipose Mesenchymal Stem Cells Carrying miRNA-22-3p Promote Schwann Cells Proliferation and Migration through Downregulation of PTEN
Source: Dis Markers. 2022 Sep 13;2022:7071877. doi: 10.1155/2022/7071877 (PMC9489425; doi:10.1155/2022/7071877)
Supplement: Supplementary Materials — Supplementary S1 Primers used in Dual-Luciferase Reporter Assays. Supplementary S2: Reaction conditions and primers used in reverse transcription and RT-qPCR. Supplementary S3: The miRNA different expression between ADSC-Exos and SC-Exos, heat map showing the dominant cluster pattern of miRNAs under p <0.05. Red signals and blue signals represent upregulated and downregulated expression, respectively. [file 7071877.f1.zip › supplementary1.doc]

Supplementary S1：

Primers used in Dual-Luciferase Reporter Assays.

Primers for plasmid construction

| **Name** | **Forward Primer 5’-3’** | **Reverse Primer 5’-3’** |
| --- | --- | --- |
| PTEN  -3`UTR-WT | TGTTTAAACGAGCTCATTTTTTTTTTCTTATCAAG | TGCCTGCAGGTCGACGAAAGTTGTCTCTGACTTTA |
| PTEN  -3`UTR-MUT | AGAAAAAATGATCGGAGTGAGTACATTTG | TCACTCCGATCATTTTTTCTAGCACCAAT |
