# Supplementary material for: Exosomes Derived from Adipose Mesenchymal Stem Cells Carrying miRNA-22-3p Promote Schwann Cells Proliferation and Migration through Downregulation of PTEN
Source: Dis Markers. 2022 Sep 13;2022:7071877. doi: 10.1155/2022/7071877 (PMC9489425; doi:10.1155/2022/7071877)
Supplement: Supplementary Materials — Supplementary S1 Primers used in Dual-Luciferase Reporter Assays. Supplementary S2: Reaction conditions and primers used in reverse transcription and RT-qPCR. Supplementary S3: The miRNA different expression between ADSC-Exos and SC-Exos, heat map showing the dominant cluster pattern of miRNAs under p <0.05. Red signals and blue signals represent upregulated and downregulated expression, respectively. [file 7071877.f1.zip › supplementary2.doc]

Supplementary S2：

Reaction conditions and primers used in reverse transcription and RT-qPCR.

1. Reverse transcription

Table 1 gDNA Erase

| Total RNA | 1ul |
| --- | --- |
| 5_X_gDNA remover | 2ul |
| ddH2O | 7μL |

Mix well, incubate at 42℃ for 2 min.

Table 2 Reverse Transcription Reaction System

| above | 10ul |
| --- | --- |
| 5_X_RT Mix | 4ul |
| Enzyme Mix | 2ul |
| Random primers | 2ul |
| ddH2O | 2μL |

Table 3 Reverse Transcription Reaction Condition

| Procedure | Temperature | Time |
| --- | --- | --- |
| Pre-Reaction | 25℃ | 5min |
| Reaction | 37℃ | 15min |
| Stop | 85℃ | 5sec |

Table 4 miRNA Reverse Transcription Primer

| **Name** | **Transreverse Primer 5’-3’** |
| --- | --- |
| miR-22-3p | GTCGTATCCAGTGCAGGGTCCGAGGTATTCGCACTGGATACGACACAGTT |

2. RT-qPCR

Table 5 RT-qPCR Reaction System

| SYBR qPCR mix | 10 ul |
| --- | --- |
| Primer F(10 um) | 0.4 ul |
| Primer R(10 um) | 0.4 ul |
| Template | 1 ul |
| ddH2O | 8.2 μL |

Table 6 RT-qPCR Reaction Condition

| Procedure | Temperature | Time |
| --- | --- | --- |
| Hold | 95℃ | 30sec |
| Denature | 95℃ | 10sec  35cycles |
| Anneal&Extend | 60℃ | 30sec |
| Stop | 85℃ | 5sec |
| End | 4℃ | +∞ |

Table 7 RT-qPCR Primers

| **Name** | **Forward Primer 5’-3’** | **Reverse Primer 5’-3’** |
| --- | --- | --- |
| PTEN | ACCAGGACCAGAGGAAACCT | TTTGTCAGGGTGAGCACAAGA |
| GAPDH | GGCTCATGACCACAGTCCAT | ACATTGGGGGTAGGAACACG |
| miRNA-22-3p | GCGAAGCTGCCAGTTGAAG | AGTGCAGGGTCCGAGGTATT |
| U6 | CTCGCTTCGGCAGCACA | AACGCTTCACGAATTTGCGT |
